# Exploring Risk Factors for Post-operative Complications in Laparoscopic Common Bile Duct Exploration: A Literature Review

**DOI:** 10.7759/cureus.72570

**Published:** 2024-10-28

**Authors:** Mina Manasseh, Islam MT Elsamalouty, Cho Nu San, Marcos Kostalas

**Affiliations:** 1 General Surgery, Torbay and South Devon NHS Foundation Trust, Torquay, GBR; 2 Upper Gastrointestinal Surgery, Torbay and South Devon NHS Foundation Trust, Torquay, GBR

**Keywords:** bile duct injury, common bile duct stones, complication, laparoscopic common bile duct exploration, outcome, risk factors

## Abstract

Gallstone-related diseases, particularly common bile duct (CBD) stones, pose a significant global health challenge. The emergence of laparoscopic common bile duct exploration (LCBDE) has transformed the management of these conditions by offering a less invasive alternative to traditional open surgery. This literature review aims to analyze published literature to identify and understand the risk factors associated with LCBDE. The study aims to offer valuable insights that could potentially enhance patient care and outcomes in managing CBD stones.

A comprehensive search of English-language studies from the past 20 years was conducted using PubMed, focusing on peer-reviewed primary research, systematic reviews, and meta-analyses. From 830 initial articles, 25 were selected based on relevance and availability. The review identified several key risk factors influencing LCBDE outcomes, including patient-related factors such as advanced age, high comorbidity scores, narrow bile ducts, and complex stones, which increase the likelihood of complications. Surgeon experience also plays a crucial role, with lower complication rates observed among surgeons who have performed at least 70 LCBDE procedures over a 10-year period. Additionally, procedure-related factors such as the transcystic (TC) approach, primary suture (PS) closure, and intraoperative imaging were found to reduce complication rates. LCBDE remains a promising approach for managing bile duct stones, particularly when patient, surgeon, and procedural factors are optimized.

## Introduction and background

Gallstone-related disorders, particularly common bile duct (CBD) stones, represent a substantial health burden worldwide. These conditions can lead to significant morbidity if left untreated, including serious complications such as cholangitis, pancreatitis, and biliary cirrhosis [1]. Historically, the management of CBD stones involved endoscopic retrograde cholangiopancreatography (ERCP) followed by a separate procedure for cholecystectomy. However, laparoscopic common bile duct exploration (LCBDE) has emerged as a transformative approach, offering a minimally invasive, single-stage solution for both CBD stone removal and cholecystectomy [2]. By allowing surgeons to directly visualize and clear the bile duct of stones while simultaneously performing a cholecystectomy, LCBDE minimizes the need for multiple interventions and reduces the risk of complications such as cholestasis and secondary biliary infections [3].

In comparison to the traditional ERCP followed by laparoscopic cholecystectomy (LC), LCBDE offers several advantages. It not only decreases the overall treatment duration and hospital stay but also reduces the cumulative procedural risk, waiting times, and healthcare costs [4]. Furthermore, LCBDE eliminates the need for two separate procedures, thus minimizing the patient's exposure to anesthesia and improving overall patient outcomes [5]. Despite its proven efficacy, complications may still occur during or after LCBDE. Common complications, such as bile leaks, infections, bile duct strictures, and pancreatitis, necessitate vigilant monitoring and management to ensure patient safety, as they are influenced by various factors [6]. Recent data from multicenter studies and systematic reviews have further confirmed the efficacy of LCBDE as a first-line treatment, particularly in complex cases. A 2023 meta-analysis comparing LCBDE with intraoperative ERCP showed that LCBDE resulted in higher stone clearance rates and lower incidences of pancreatitis, positioning it as the globally preferred technique in many high-volume centers [7,8].These findings highlight the increasing prominence of LCBDE as a safer, more efficient alternative to the traditional two-stage approach.

Understanding the risk factors associated with these complications is crucial for improving surgical outcomes and patient safety. Risk factors in LCBDE can be broadly classified into three categories: patient-related factors (e.g., age, co-morbidities, and body mass index), surgeon-related factors (e.g., experience and technical skill), and procedure-related factors (e.g., complexity of the CBD stones and duration of the procedure) [9]. This review aims to provide a comprehensive synthesis of the existing literature on risk factors contributing to post-operative complications in LCBDE, with the goal of enhancing the clinical management of patients undergoing this procedure and improving overall outcomes.

## Review

Search strategy 

Keywords

“Laparoscopic common bile duct exploration”, and/or “Complication”, and/or “risk factors”, and/or “Postoperative complications” and/or, “Co-morbidity” and/or, “High BMI” and/or, “Bile Leak”.

*Data Collection* 

Data collection involved a comprehensive search for primary research studies, systematic reviews, and meta-analyses pertaining to risk factors for complications following LCBDE. The search was conducted on PubMed, focusing on English-language articles published between 2004 and August 2024. and encompassing all age groups. Peer-reviewed journal articles were prioritized. All relevant studies reporting complications after laparoscopic CBD exploration were eligible for inclusion if available as full texts, structured abstracts, or conference reports. Exclusion criteria comprised studies describing procedures conducted through open surgery, non-English articles, articles lacking full-text accessibility, or those addressing procedures other than CBD exploration. The initial search yielded 830 articles. This refined approach ensured a comprehensive, yet targeted selection of studies aligned with the scope of our investigation. A total of 25 records were identified by the literature search. 

Results 

**Table 1:** List of studies included evaluating risk factors for complications in LCBDE LCBDE: Laparoscopic common bile duct exploration

Study ID	Authors	Year	Type of Study	Level of Evidence
1	Zhu et al. [10]	2020	Retrospective Cohort Study	III
2	Parra-Membrives et al. [11]	2019	Retrospective Cohort Study	III
3	Kao et al. [12]	2021	Multicenter Retrospective Study	III
4	Yeon et al. [13]	2022	Comparative Study	III
5	Jia et al. [14]	2020	Retrospective Cohort Study	III
6	Zerey et al. [15]	2018	Guidelines/Review	V
7	Xie et al. [16]	2023	Multicenter Retrospective Study	III
8	Lucocq and Nassar [17]	2024	Retrospective Cohort	III
9	Zhu et al. [18]	2018	Retrospective Cohort	III
10	Li et al. [19]	2020	Retrospective Cohort	III
11	Wang et al. [20]	2022	Retrospective Cohort	III
12	Nassar et al. [21]	2022	Cohort Study	II
13	Liu et al. [22]	2017	Retrospective Cohort	III
14	Ma et al. [23]	2022	Retrospective Cohort	III
15	Hodgson et al. [24]	2021	Cohort Study	II
16	Wahi et al. [25]	2023	Retrospective Study	III
17	Hajibandeh et al. [26]	2019	Systematic Review and Meta-analysis	I
18	Al-Ardah et al. [27]	2021	Retrospective Study	III
19	Ahmed and Redwan [28]	2020	Retrospective Cohort	III
20	Ma and Cai [29]	2023	Meta-analysis	I
21	Zhu et al. [30]	2021	Meta-analysis	I
22	Yin et al. [31]	2022	Retrospective Cohort	III
23	Wang et al. [32]	2022	Retrospective Cohort	III
24	Altieri et al. [33]	2018	Retrospective Study	III
25	Bush et al. [34]	2022	Retrospective Study	III

In the context of LCBDE, complications are influenced by several critical factors that can be broadly categorized into three groups: patient-related, surgeon-related, and procedure-related factors.

Patient-Related Risk Factors

Patient-related factors significantly influence the likelihood of complications during LCBDE. These include age, comorbidities, obesity, CBD size, difficult stones, and abnormal liver function tests (LFTs). Each of these factors requires thorough consideration.

Age: Advanced age is a well-established risk factor for complications in LCBDE, largely attributed to decreased physiological reserves, increased comorbidities, and higher American Society of Anesthesiologists (ASA) grading. Older adults are particularly vulnerable to pulmonary complications, as demonstrated by Zhu et al., who reported a significant odds ratio of 4.41 (95% CI 1.78-10.93; p=0.001) for respiratory issues compared to younger patients [10]. This suggests that while LCBDE is generally safe, careful perioperative monitoring is crucial for elderly patients to mitigate the risk of respiratory complications.

Additionally, age has been linked to a higher recurrence of choledocholithiasis following LCBDE. Parra-Membrives et al. observed a 14.1% recurrence rate among patients over 55 years (mean age 65.35), indicating that age increases the likelihood of stone recurrence, potentially due to decreased bile flow or other age-related physiological changes [11]. Similarly, Kao et al. found that older patients (mean age 61) were more likely to experience unsuccessful LCBDE procedures (p=0.002), reinforcing the challenges posed by age in achieving successful outcomes [12].

In a broader analysis by Yeon et al., which included 363 patients divided into two groups (<80 years and ≥80 years), the older cohort exhibited higher Charlson Comorbidity Index scores (≥5) and ASA grades (≥3), indicating greater baseline risks [13]. Despite these risks, complication rates did not significantly differ between the two age groups, suggesting that factors such as surgical technique and postoperative care may play a critical role in mitigating age-related risks. The study also identified a Charlson Comorbidity Index ≥5 (OR=2.307; 95% CI 1.162-4.579; p=0.017) and prolonged operative times (>2 hours) as significant predictors of complications. This underscores the importance of optimizing preoperative evaluation and minimizing operative time in older patients to reduce complication rates.

Comorbidity: Pre-existing conditions such as diabetes, hypertension, coronary heart disease, and liver disease can significantly increase the risk of complications during LCBDE. These comorbidities affect surgical outcomes by impairing wound healing, increasing the risk of infections, and complicating recovery. For instance, diabetic patients may experience delayed healing, while liver disease can disrupt normal clotting and elevate bleeding risks.

Jia et al. investigated 98 patients undergoing emergency LCBDE within eight hours of admission and found that, despite comorbidities like coronary heart disease and diabetes, the procedure's success rates remained consistent across patients with varying ASA grades (II, III, and IV) [14]. The study highlighted minimal complications and a low recurrence rate of CBD stones, indicating that LCBDE can be performed safely in high-risk patients if appropriate preoperative planning and intraoperative management are employed.

Supporting this, a review by the Society of American Gastrointestinal and Endoscopic Surgeons (SAGES) emphasized that while LCBDE is generally safe, patients with comorbidities are at an elevated risk of postoperative issues such as infections, bile leaks, or cardiovascular events [15]. However, careful monitoring and surgical expertise can mitigate these risks. Additionally, a multicenter study published in Langenbeck’s Archives of Surgery confirmed that LCBDE remains effective in patients with complex conditions, including diabetes and cardiovascular disease, with only a slight increase in wound infections among obese patients [16]. This reinforces that with skilled surgical management, LCBDE can be safely applied to a diverse patient population despite underlying health conditions.

Previous Abdominal Biliary Tract Surgery: The presence of prior abdominal surgeries, particularly open or upper gastrointestinal procedures, poses significant challenges for LCBDE due to the development of adhesions and alterations in anatomical structure. These complications contribute to increased technical difficulty, resulting in prolonged operative times and a heightened risk of access-related complications. In a comprehensive study involving 5,916 patients who underwent LC and LCBDE, it was found that 1,846 (31.2%) had previously undergone abdominal surgeries. These patients demonstrated a greater need for adhesiolysis, particularly in the duodenal and hepatic flexure regions, and experienced higher rates of bowel injury associated with access (0.4% vs. 0.0%, p<0.001) [17]. Among those with a history of upper gastrointestinal and biliary surgeries, the incidence of adhesiolysis was notably high (76.3%), along with a considerable degree of procedural difficulty (64.9% rated grades 3-5), often necessitating specialized techniques like fundus-first dissection (p<0.05). Furthermore, the findings indicated that patients with a history of open surgery had longer operative durations compared to those who had undergone laparoscopic procedures (65 vs. 55 minutes, p<0.001).

Supporting these results, a retrospective study conducted by Zhu et al. in 2018 assessed 217 patients with CBD stones, comparing outcomes between those with (Group A) and without (Group B) a history of upper abdominal surgery [18]. Group A experienced longer operative times (179.7 ± 61.5 vs. 156.0 ± 46.8 minutes, p=0.014), yet no significant differences were observed regarding blood loss, post-operative hospital stays, complication rates, or stone clearance success between the two groups. Notably, both groups achieved comparable final stone clearance rates (100%) and exhibited low stone recurrence rates (2.0% vs. 2.4%, p=1.000), reinforcing the safety and efficacy of LCBDE even in patients with previous abdominal surgeries. Collectively, these studies highlight the critical role of surgical expertise and preoperative planning in addressing the complexities associated with prior surgical history. Although adhesions and anatomical variations complicate LCBDE, careful adhesiolysis and specialized techniques can facilitate successful outcomes with minimal complications.

In addition, a study by Li et al. in 2020 examined 227 patients with a history of abdominal biliary surgeries and found that those undergoing LCBDE experienced fewer complications than those who had open common bile duct exploration (OCBDE) (p=0.045) [19]. The research indicated that patients with fewer than two prior surgeries or a background of laparoscopic surgery achieved better outcomes, thus positioning LCBDE as a safer and less invasive option in such cases.

High BMI: Obesity is a well-recognized risk factor in many surgical procedures, LCBDE, where it can add technical challenges, especially during suturing after choledochotomy. Wang et al. observed that a higher BMI can prolong suture time due to increased difficulty during the procedure [20]. Specifically, their study found that a higher BMI (r=0.486, p<0.0001) and previous biliary tract surgeries (r=0.384, p<0.0001) were correlated with prolonged suture times, highlighting the complexity involved in handling these cases.

In contrast, Nassar et al., who analyzed data from 683 patients with an average BMI of 39.9, found that while obesity did not significantly increase operative difficulty or complication rates compared to non-obese patients, there was a slightly higher rate of wound infections among the obese group (1.9% vs. 0.7%; p=0.002) [21]. However, other metrics such as operative time, morbidity, and readmission rates remained comparable between the two groups, suggesting that despite the technical challenges posed by obesity, the outcomes of LCBDE are not significantly compromised. Importantly, the study emphasized that refining surgical techniques, particularly for access and closure, can mitigate some of the difficulties associated with higher BMI, and patients should not be denied surgery based solely on their weight.

Together, these studies underscore that while obesity can present additional challenges during LCBDE, careful surgical planning and technique adjustments allow for comparable outcomes to those in non-obese patients, making the procedure feasible and effective regardless of BMI.

CBD Size: A narrow CBD is identified as a significant risk factor for bile leaks following primary closure after LCBDE. In a retrospective cohort study conducted from February 2012 to June 2016, a total of 265 LCBDE procedures were analyzed, with 141 patients receiving primary closure. Among these patients, bile leakage was documented in 11.3% (16 out of 141). The occurrence of bile leaks was significantly higher in individuals with a slender CBD, where leakage rates reached 31.6% for those with a CBD diameter of less than 1 cm, compared to just 7.0% for those with a diameter of 1 cm or greater (p = 0.04) [22]. These results underscore the importance of CBD size as a key risk factor for bile leaks after primary closure following LCBDE.

Difficult Stones: The management of challenging stones, such as multiple duct stones, poses a significant risk for complications during LCBDE. Kao et al. evaluated 513 patients undergoing LCBDE and found the presence of multiple stones was associated with a markedly higher risk of severe complications, with an odds ratio of 3.79 (95% CI: 1.66-8.67, p=0.002) [12]. Interestingly, the study also suggested that patients with multiple stones had a lower likelihood of operation failure compared to those with a single impacted stone, though this observation lacked statistical significance.

Ma et al. conducted a comprehensive study of 334 patients with difficult biliary stones, characterized by stones that were large (>15 mm), multiple (>3), intrahepatic, or impacted [23]. Their findings revealed an impressive 98.8% overall stone clearance rate, with no occurrences of bile duct injury, perforation, or surgery-related mortality. Post-operative complications, such as bile leakage, were reported in 4.8% of patients, all of which were successfully managed with conservative treatments.

Deranged LFTs: Abnormal LFTs, particularly non-bilirubin markers, are significant predictors of LCBDE outcomes. Elevated LFTs can indicate underlying liver dysfunction, which may complicate the procedure and increase the likelihood of a failed operation. In a study by Kao et al., patients who experienced unsuccessful LCBDE had notably higher rates of abnormal LFTs compared to those with successful outcomes (53.2% vs. 37.1%; p=0.031). This emphasizes the critical role of preoperative LFT evaluation in predicting procedural success and guiding surgical decisions [12].

*Surgeon-Related Risk Factors: Experience and Surgical Load* 

Surgeon experience and surgical workload are the most significant factors influencing the outcomes of LCBDE procedures. A study by Liu et al. demonstrated that in a cohort of 141 patients who underwent primary closure following LCBDE, 11.3% experienced bile leakage [22]. This complication was more common in patients with a narrower CBD (<1 cm vs. ≥1 cm: 31.6% vs. 7.0%, p=0.04) and in cases managed by less experienced surgeons (early cases vs. later cases: 17.1% vs. 5.6%, p=0.04). Multivariable regression analysis identified both a narrow CBD (OR: 3.799 (95% CI: 1.081-13.349), p=0.04) and surgeon experience (OR: 4.228 (95% CI: 1.330-13.438), p=0.03) as independent predictors of bile leakage. Notably, surgeon experience emerged as the most influential factor, with bile leakage rates declining from 15.7% in earlier cases to 7.0% in later ones as surgeons gained proficiency.

While specialists in LCBDE tend to have higher success rates, non-specialist surgeons can achieve comparable outcomes. Hodgson et al. found that though specialists had a higher success rate (90.8% versus 82.6%, p = 0.008), they also had longer operative times [24]. Their study suggests that achieving an 80% success rate requires performing at least 70 LCBDE procedures over a 10-year span, reinforcing the relationship between procedural volume and surgical success.

The British Benign Upper Gastrointestinal Surgical Society (BBUGSS) has established key performance indicators (KPIs) for LCBDE, which include maintaining conversion rates below 10%, achieving duct clearance rates above 85% (with a target of over 90%), and limiting bile leak rates to less than 5% [35]. These standards emphasize the importance of experience and case volume in ensuring favorable outcomes for patients undergoing LCBDE.

*Procedure-Related Risk Factors* 

Procedure-related risk factors play a pivotal role in the complications associated with LCBDE. These factors encompass the type of approach, choice between primary suture (PS) and T-tube drainage (TTD), and the utilization of intraoperative cholangiography (IOC).

Type of Approach: Stone removal during LCBDE can be accomplished via different approaches, including the transcystic (TC) and transductal (TD) routes. The TC approach is considered both effective and safe for treating CBD stones, as shown by Wahi et al. in 2023 [25]. It is generally preferred over the trans-choledochal approach due to a lower risk of complications, particularly bile leaks. In a 2019 systematic review by Hajibandeh et al. 2019, involving 4,073 patients (2,176 treated via the TC route and 1,897 via the TD route) [26]. The TC approach was linked to a lower overall complication rate (p=0.001) and fewer biliary complications (p=0.0003) compared to the TD approach. Furthermore, patients undergoing the TC method experienced less blood loss , shorter hospital stays, and reduced procedure times. However, both approaches demonstrated no significant difference regarding rate of CBD clearance (p=0.77) and conversion to open surgery (p=0.86).

In another retrospective study conducted by Al-Ardah et al., the outcomes of the two approaches for managing choledocholithiasis were compared across 200 cases [27]. Out of 179 patients who had stones confirmed, 111 were treated using the TC route, while 68 underwent the TD approach; notably, 25% of the TD cases had initially been attempted via the TC route but required conversion. The study found that the TC route is associated with lower complication rates and shorter hospital stays compared to the TD approach, reinforcing its preference as a treatment strategy for choledocholithiasis.

Interestingly, a study by Ahmed et al. evaluated the impact of different choledochotomy techniques-scalpel, scissors, diathermy hook, or ultrasonic device-on complication rates during LCBDE [28]. The study, which involved 85 patients with choledocholithiasis, found no significant differences in short- and long-term outcomes among the various methods used

PS vs. TTD: Following a choledochotomy, the CBD can either be closed primarily with sutures or managed with TTD to reduce the risk of postoperative complications such as biliary fistula and strictures. A 2023 meta-analysis by Ma et al. assessed 1,549 patients: 827 in the PS group and 722 in the TTD group [29]. The analysis revealed that the PS group experienced significantly fewer postoperative complications compared to the TTD group (p=0.006). Although no notable differences were observed between the groups in terms of bile leakage (p=0.326) or bile duct stricture (p=0.679), the PS group benefited from shorter operative times (p ≤ 0.001) and less intraoperative bleeding. Overall, the PS group demonstrated better outcomes, including shorter hospital stays and fewer postoperative complications.

Similarly, Zhu et al. analyzed data from 1,865 patients across six randomized controlled trials (RCTs) and 10 cohort studies to compare primary duct closure (PDC) with TTD after LCBDE [30]. The RCT results showed that the PDC group had shorter operation times, fewer postoperative complications, reduced hospital stays, and lower hospitalization costs (all p<0.05). Findings from the cohort studies also supported these benefits, showing that PDC was linked to less intraoperative blood loss and fewer overall complications. However, the rates of bile leakage, retained stones, stone recurrence, bile duct stricture, and other postoperative complications did not differ significantly between the two groups.

Further supporting these conclusions, Yin et al. compared PS and TTD in a retrospective study focused on patients with secondary CBD stones [31]. Their results indicated that the PS group had shorter operation times, reduced hospital stays, and fewer complications. In a similar study, Wang et al. confirmed that PDC following choledochotomy was both safe and effective, particularly for managing acute cholangitis caused by CBD stones [32]. These studies collectively reinforce the growing preference for primary closure as a safer alternative to TTD, offering reduced complication rates and enhanced patient recovery.

Usage of Intraoperative Imaging: IOC plays a crucial role in visualizing the bile ducts during surgery, aiding in the identification of anatomical variations and minimizing the risk of bile duct injuries. Despite its value, the use of IOC in LC has decreased in the US, a trend that has coincided with a rise in bile duct injuries [33]. This decline raises concerns about the implications of omitting intraoperative imaging in procedures that involve complex biliary anatomy.

Recent studies highlight the benefits of intraoperative imaging in managing choledocholithiasis. For instance, a 2022 study at Torbay Hospital examined 506 patients undergoing LC for gallstones who showed markers of choledocholithiasis but lacked preoperative confirmation. Intraoperative imaging revealed that 65.6% of patients had clear bile ducts, while 142 patients were found to have stones and subsequently underwent LCBDE, achieving a 95.8% success rate. The sensitivity and specificity of this approach were 93.3% and 99.1%, respectively, indicating its high diagnostic accuracy. The study concluded that intraoperative imaging, combined with immediate stone removal, provides an effective "single-stage" solution for managing choledocholithiasis, reducing the need for additional procedures [34].

Further supporting these findings, another 2022 study from the same institution assessed 311 patients undergoing LCBDE, with a 94% completion rate using laparoscopic methods. The incidence of bile leakage was low at 4.2%, and retained stones within 90 days occurred in only 3.9% of cases. These results emphasize that LCBDE, when combined with intraoperative imaging, is not only safe but also highly effective in preventing complications. The reproducibility of these outcomes across a national scale highlights the potential of this approach to become a standard practice for the management of bile duct stones [36].

Discussion

Our review of the literature has underscored the multifaceted role of LCBDE in the management of CBD stones. As a minimally invasive technique, LCBDE offers clear advantages over traditional treatments, such as open surgery or two-stage procedures involving ERCP. Its increasing use reflects its value in addressing the complexities of gallstone-related disease.

The analysis of risk factors associated with LCBDE complications highlights the critical role of patient-specific, surgeon-specific, and procedure-specific factors. Patient-related risks, such as advanced age, comorbid conditions, and complex anatomical variations, significantly influence outcomes. For instance, elderly patients or those with coexisting health conditions are more susceptible to postoperative complications, requiring careful preoperative evaluation to optimize outcomes.

Surgeon expertise also plays a pivotal role in LCBDE success. The findings emphasize the importance of surgical skill and experience in ensuring successful outcomes within national healthcare systems like the NHS, demonstrating that LCBDE is both reproducible and effective at scale.

Procedure-related factors also significantly impact the success of LCBDE. Critical aspects such as the choice between TC and TD approaches, closure methods, and the routine use of intraoperative imaging influence complication rates. For instance, the TC approach is associated with fewer complications and faster recovery, while PDC, rather than traditional TTD, has been shown to reduce postoperative morbidity and hospital stays. These procedural decisions play a key role in optimizing the safety and efficacy of LCBDE.

While LCBDE shows great promise, certain limitations in the current literature should be acknowledged. One notable gap is the limited exploration of gender-based differences in LCBDE outcomes. Future research is needed to investigate whether men and women experience different complications or recovery patterns, which could help tailor surgical approaches to specific demographics. Additionally, the heterogeneity across studies in terms of design, patient selection, and follow-up duration makes it difficult to draw definitive conclusions. Standardizing methodologies in future research could enhance comparability and reliability.

Another significant limitation is the lack of long-term follow-up in many studies. This hinders a comprehensive understanding of the durability of LCBDE’s success, particularly concerning late complications like bile duct strictures or the need for further intervention. Long-term data would provide valuable insights into the sustainability of LCBDE as a first-line treatment for CBD stones.

Despite these limitations, the body of evidence supports the continued expansion of LCBDE, particularly as surgeon expertise grows and procedural refinements continue. The strong correlation between surgeon experience and positive outcomes suggests that LCBDE should be concentrated in high-volume centers or performed by surgeons with specialized training. Establishing structured training programs and mentorship for surgeons could further enhance procedural success.

Additionally, growing support for the TC approach, PS closure, and the integration of intraoperative imaging suggests that these techniques should be incorporated into standard practice where feasible. These advancements could help reduce complication rates, shorten hospital stays, and improve patient outcomes on a larger scale.

## Conclusions

LCBDE is a promising option for managing CBD stones, particularly when performed in high-volume centers by skilled surgeons. The success and utility of LCBDE can be significantly enhanced through the meticulous optimization of patient-related, surgeon-related, and procedure-related factors. By addressing and fine-tuning these key elements, the occurrence of complications associated with LCBDE can be mitigated, thereby reinforcing its potential as a primary choice for managing various bile duct conditions. This strategic approach not only underscores the clinical efficacy of LCBDE but also advocates for its expanded utilization as a frontline intervention in the comprehensive management of bile duct disorders.
